# Aqueous Cytokine Changes Associated with Posner-Schlossman Syndrome with and without Human Cytomegalovirus

**DOI:** 10.1371/journal.pone.0044453

**Published:** 2012-09-13

**Authors:** Jing Li, Marcus Ang, Chui Ming Gemmy Cheung, Maya Vania, Anita Sook Yee Chan, Samanthila Waduthantri, Henry Yang, Soon Phaik Chee

**Affiliations:** 1 Department of Ophthalmology, Xinhua Hospital, Shanghai Jiao Tong University School of Medicine, Shanghai, People’s Republic of China; 2 Ocular Inflammation Group, Singapore Eye Research Institute, Singapore, Singapore; 3 Ocular Inflammation and Immunology Services, Singapore National Eye Centre, Singapore, Singapore; 4 Cancer Science Institute of Singapore, Singapore, Singapore; 5 Department of Ophthalmology, Yong Loo Lin School of Medicine, National University of Singapore, Singapore, Singapore; 6 Department of Pathology, Singapore General Hospital, Singapore, Singapore; The Ohio State University, United States of America

## Abstract

**Aim:**

To study the differences in aqueous cytokines in Posner-Schlossman Syndrome (PSS) patients with and without human cytomegalovirus (CMV) DNA in the aqueous humor.

**Methods:**

This is a prospective study. Fifty-three uveitis patients with clinical signs of PSS were enrolled and aqueous humor samples were collected. Fourteen PSS patients were positive of CMV DNA in the aqueous by polymerase chain reaction (PCR) analysis. These eyes were negative of common ocular pathogens such as herpes simplex virus, varicella-zoster virus, rubella virus and toxoplasma. Twenty-five otherwise healthy cataract patients were enrolled as controls. Cytokine concentration was measured by a magnetic color-bead-based multiplex assay and analyzed using statistical and classification approaches.

**Results:**

The average age of 53 PSS patients was 48.74±13.43 years (yrs) (mean ± standard deviation) and 66.3±15.0 yrs for the controls. The median CMV viral DNA copy number was 26000/mL aqueous (range 1400 to 85000 copies/mL) in 14 CMV positive patients as determined by quantitative PCR. PSS aqueous had significantly higher Interleukin (IL)-8 (CXCL8), monocyte chemotactic protein-1 (CCL2), macrophage inhibitory protein 1-β (CCL4), granulocyte colony-stimulating factor (GCSF) and transforming growth factor-β (TGF-β) levels than controls after adjusted by age and gender. IL-2, IL-12, tumor necrosis factor-α (TNF-α) and interferon-α (IFN-α) levels were significantly lower in PSS aqueous than controls. No difference between CMV positive PSS and CMV negative PSS aqueous was observed. Over 97% of PSS samples were distinguished from controls by elevated CXCL10 (>500 ng/mL), CXCL8 (>30 ng/mL) and CCL2 (>60 ng/mL) levels.

**Conclusion:**

PSS eyes were characterized by elevated aqueous chemokine concentration. The presence of CMV viral DNA was not associated with significant change of the type of cytokine expression in PSS patients.

## Introduction

It is estimated that about one-third of anterior uveitis is of unknown etiology despite having distinctive clinical features. Among those, Posner-Schlossman Syndrome (PSS) or glaucomatocyclitic crisis is typically characterized by recurrent episodes of elevated intraocular pressure (IOP), mild anterior chamber inflammation with a few keratic precipitates and rarely, heterochromia with anisocoria. [1] In the majority of cases, the condition is unilateral and patients retain a normal visual field and optic disc. However the rise of IOP is often out of proportion to the severity of the inflammation. Thus, PSS may be misdiagnosed as acute or chronic angle closure glaucoma or even as Fuchs heterochromic iridocyclitis (FHI).

Human cytomegalovirus (CMV) is an opportunistic virus which establishes a lifelong latent infection in endothelial cells, macrophages and granulocyte progenitor cells following the primary infection [2]. The seroprevalence of CMV infection is high worldwide, reaching 100% in underdeveloped regions [3]. CMV reactivation is a major cause of mortality in patients with compromised immune response, such as in HIV patients, organ transplant recipients and patients on immunosuppressive medications. In recent years, reactivation of CMV in immunocompetent humans has been increasingly recognized [4]–[6]. Such reactivation is usually limited to inflamed tissues and certain cancers. CMV reactivation in immunocompetent patients have been associated with stress [7], chronic inflammation [8], [9] and the use of steroid [10], [11].

A number of studies have reported the identification of human CMV viral DNA in aqueous samples from patients with anterior uveitis and endotheliitis [12]–[24]. Our previous study showed that up to 52.2% of PSS patients were positive of CMV viral DNA as determined by quantitative PCR analysis [15]. In the context of high seroprevalence of human CMV infection, these findings suggest the possibility that intraocular human CMV reactivation or infection may be associated with the etiology of idiopathic uveitis in immunocompetent patients. In this study, we compared the aqueous cytokine profiles in immunocompetent PSS patients with and without CMV viral DNA.

## Patients and Methods

### Patient Eligibility and Recruitment

This is a prospective study performed in accordance to the tenets of the Declaration of Helsinki and approved by the Institutional Review Board (IRB) of Singapore National Eye Centre. Written consent was obtained from each participating patient.

Consecutive patients with clinical signs of Posner-Schlossman Syndrome presenting to the Singapore National Eye Centre (SNEC) Ocular Inflammation and Immunology Service from 1^st^ January 2008 to 1^st^ January 2010 were enrolled in the study. The inflammation were analyzed using the criteria set by the Standardization of Uveitis Nomenclature (SUN) working group for scoring the anatomical location, onset, duration, course and activity. [25] The clinical criteria for the diagnosis of PSS are: recurrent elevated IOP higher than 21 mmHg, mild anterior chamber inflammation (anterior chamber cells: occasional to 1+SUN grade) with fine to medium keratic precipitates and unilateral eye involvement. In addition, all patients were immunocompetent. A complete clinical evaluation of each patient excluded the possibility of known systemic, genetic and infectious causes for the inflammation. Specifically, all patients were negative for tuberculosis, sarcoidosis, syphilis and herpes simplex virus, varicella-zoster virus, rubella virus and toxoplasmosis genomic DNA in the aqueous samples by PCR as described previously [14]. Patient demographic information was recorded. PCR analysis for human CMV virus was performed by PCR as previously described. [14].

Twenty-three patients elective for cataract surgery were enrolled as controls for the study during the same study period. These patients had no known systemic inflammatory, autoimmune or immunosuppressive disease, no pre-existing ocular disease or previous ocular surgery.

### Aqueous Humor Sampling

In eyes with PSS, aqueous humor was collected in the clinic before the commencement of treatment. [16] Patients were given topical anesthesia and a 27-gauge insulin syringe was inserted at the peripheral clear cornea in a plane above and parallel to the iris. Under direct vision aided by the slit-lamp, approximately 100 µL of aqueous fluid was withdrawn. All patients were re-examined at 1 week after the procedure, with topical antibiotics prescribed for 1 week. For cataract controls, aqueous humor was aspirated into a sterile insulin syringe at the beginning of cataract surgery after paracentesis was performed.

All samples were spun at 10000 rpm for 5 minutes before they were stored in −80°C until further analysis.

### Cytokine Analysis

Bio-Plex Pro™ magnetic colour-bead-based multiplex assay (Bio-Rad Laboratories, Inc., Hercules, CA, USA) was used to measure the concentrations of cytokines/chemokine. The assay was conducted according to the manufacturer’s instruction. Thirty-five microliters (35 µL) of aqueous humour sample was used in each reaction [26]. Fluorescence intensity (FI) from the immunoassay was acquired and analyzed using Bio-Plex™ 200 System (software version 6.0, Bio-Rad Laboratories, Inc., Hercules, CA). Samples with calculated concentrations lower than the low detecting limit for individual cytokine were defined as non-measurable for the specific cytokine.

### Classification Analysis

Stepwise analysis was performed to generate cytokine profiles to best separate PSS aqueous from cataract samples as described previously. (Zhou et al, 2008) Prior to the classification, all measured concentrations were logarithmically transformed and a Student t test was performed to determine the significance of the mean difference between two groups (PSS and cataract) for each cytokine. Cytokines with high p value (p>0.05) were removed from the panel. At the first classification step, all PSS and cataract cytokine data were employed with a linear classifier. The goal was to robustly and confidently identify a subgroup of PSS patients with high cytokine concentrations. The cytokine with 100% accuracy and the highest concentration margin between PSS and cataract group was chosen (CXCL10). The remaining PSS patient samples and all cataract samples were then subjected to a second classification step and a group of cytokines were identified in order to achieve the highest classification efficiency. This panel of cytokines was further analyzed and cytokines which showed high degrees of correlation to others in the panel was removed on condition that the removal did not reduce the classification efficiency.

The classification efficiency was evaluated by the following formula: the number of true subjects (based on clinical diagnosis) divided by the total number of subjects within the group represented in percentage.

### Statistical Analysis

Statistical analysis was performed using Statistical Package for the Social Sciences (SPSS) Version 19 (IBM Corporation, Armonk, NY). Statistical significance was accepted at *p*≤0.05. Age difference was compared by one way ANOVA analysis. Gender, ethnic group differences and the differences in percentage of samples with measurable concentrations for each cytokine were compared by Pearson χ^2^ analysis. For the differences in cytokine concentrations, Mann-Whitney U test with Bonferroni correction, and binary logistic regression analysis including age and gender variation were performed. Due to the multicolinearity among cytokines, the binary logistic regression analysis was performed with individual cytokine.

## Results

### Patient Demographics and Clinical Presentation

Fifty-three patients who were diagnosed with PSS were enrolled in this study during this time period. The demographics of the patients are summarized in Table 1. The mean age of PSS patients was 54.6 years (yrs) (range 24 to 72 yrs). There were 23 males (43.4%) and 30 females (56.6%).

**Table 1:** Demographic data of PSS and control patients.

		Groups			
	All (n = 76)	CMV+ PSS (n = 14)	CMV- PSS (n = 39)	Cataract (n = 23)	p-vlaue
Age, years (mean±SD)	54.6±15.7	49.7±14.3	48.4±13.2	66.3±14.99	<0.001*
Gender (%)					
Male	34 (44.7)	10 (71.4)	13 (33.3)	11 (47.8)	0.046^†^
Female	42 (55.3)	4 (28.6)	26 (66.7)	12 (52.2)	
Race (%)					
Chinese	67 (88.2)	13 (92.9)	35 (89.7)	19 (82.6)	0.755^†^
Malay	5 (6.6)	1 (7.1)	2 (5.1)	2 (8.7)	
Indian	3 (3.9)	0 (0.0)	2 (5.1)	1 (4.3)	
Others	1 (1.3)	0 (0.0)	0 (0.0)	1 (4.3)	

CMV: Cytomegalovirus, PSS: Posner-Schlossman Syndrome, CMV+PSS: PSS patients with positive CMV viral DNA in the aqueous detected by PCR, CMV-PSS: PSS patients with no human CMV DNA detected by PCR. *p-value by one-way ANOVA test, †p-value by Pearson X^2^ test.

Among PSS patients, fourteen were positive of CMV DNA in the aqueous by either conventional (n = 12) or quantitative PCR analysis (n = 14) or both. They were thus grouped as CMV positive PSS (CMV+PSS). The rest 39 samples were negative of CMV DNA and were grouped as CMV-PSS. The median CMV copy number for the 14 CMV+PSS patients was 26000/mL aqueous as determined by quantitative PCR with a range of 1400 to 85000 copies/mL. There were 10 male patients in the CMV+PSS group (71.4%) and 13 male patients in the CMV-PSS group (33.3%).

Twenty-three healthy subjects with no ocular condition other than cataract were recruited as controls. The mean age of the control group was 66.3 yrs (range 25 to 82 yrs). The control group was significantly older than the PSS group (p = 0.046, Pearson X^2^ test). There were 11 males (47.8%) and 12 females (52.2%) in this group.

### Statistical Analysis of Cytokines between PSS and Controls

Table 2 shows the median and range of concentrations for 21 cytokines measured in CMV+PSS, CMV-PSS and control aqueous samples. The number of samples which gave measurable concentrations for each cytokine in each group is also listed in Table 2.

**Table 2:** Cytokine concentrations in PSS and control aqueous samples.

Cytokines	CMV+PSS		CMV–PSS		Controls		LOD^a^
	n = 14		n = 39		n = 23		
	Median (Range)	Measurable/Total	Median (Range)	Measurable/Total	Median (Range)	Measurable/Total	
IL-2*	0.0 (0–10)	2/14†	0.0 (0–9)	4/39†	2.8 (0–17)	17/23	1.6
IFN-γ	0.0 (0–57)	4/14	0.0 (0–420)	13/39	12.7 (0–91)	12/21	6.4
IL-4	0.0 (0–2)	2/14	0.0 (0–6)	12/39	0.3 (0–6)	4/23	0.7
IL-5*	0.4 (0–5)	5/14†	0.3 (0–15)	16/39†	0.0 (0–5)	1/23	0.6
IL-13	0.6 (0–8)	6/14†	0.6 (0–14)	17/39†	3.6 (0–8)	20/23	0.7
IL-17	0.8 (0–25)	4/14	0.0 (0–34)	9/39	2.3 (0–11)	8/21	3.3
IL-6*	12.1 (1–384)	12/14	15.0 (0–1494)	30/39†	2.2 (0–14)	8/22	2.6
IL-10	1.1 (0–13)	10/14	0.5 (0–49)	22/39†	1.5 (0–5)	20/23	0.3
IL-12*	0.0 (0–17)	2/14†	0.0 (0–7)	3/39†	8.7 (0–27)	19/23	3.5
IL-1β	0.2 (0–4)	4/14	0.3 (0–5)	16/38	0.6 (0–3)	12/22	0.6
TNF-α*	0.0 (0–44)	2/14†	0.0 (0–39)	2/39†	9.6 (0–34)	17/23	6.0
IFN-α*	12.6 (0–27)	9/12	0.0 (0–118)	18/38†	30.8 (0–139)	19/23	4.3
CCL2*	401.1(174–3665)	14/14	459.9 (0–4030)	38/39	180.9 (32–962)	23/23	1.1
CCL4*	46.1(10–209)	14/14	39.6 (0–612)	38/39	17.1 (4–137)	23/23	2.4
CXCL8*	24.0 (2–200)	14/14	23.5 (0–481)	37/39	3.0 (1–20)	21/22	1.0
CXCL9^b^	0.2 (0–42.8)	12/12	0.14 (0–282)	38/36	0.06 (0–0.83)	19/21	1.2
CXCL10^b^	11.8 (0–273.2)	12/14	9.0 (0–1154.8)	25/39	0.06 (0–0.77)	20/22	6.1
IL-7	2.0 (0–23)	9/14	2.7 (0–31)	25/39†	1.1 (0–8)	8/22	1.1
G-CSF*	0.3 (0–33)	5/14†	0.0 (0–36)	11/39†	0.0 (0–9)	1/23	1.7
GM-CSF*	0.0 (0–217)	5/14†	0.0 (0–1325)	13/39†	139.6 (0–766)	20/23	2.2

All concentrations were at the unit of pg/mL except for CXCL9 and CXCL10, which were at ng/mL. LOD: low limit of detection (pg/mL) as given by Bio-Rad. B: concentrations at ng/mL,

* : p≤0.05 by Mann-Whitney U test with Bonferroni correction for cytokine concentations, PSS (including bot CMV+ and CMV- groups) compared to control group.

† : p≤0.05 by X^2^ test, PSS compared to controls for the number of samples with measureable concentrations out of total samples measured.

When CMV+PSS and CMV-PSS groups were compared to controls, the percentage of measurable samples for cytokines IL-2, IL-13, IL-12, TNF-α and GM-CSF were significantly lower in the PSS groups (p≤0.05, X^2^ test). On the other hand, the percentage of measurable samples for cytokines IL-5, IL-6 and G-CSF were significantly higher in the PSS groups than the controls (p≤0.05, X^2^ test). The percentage of measurable samples for IFN-α and IL-7 in CMV-PSS group was also significantly higher than the control group.

We then performed Mann-Whitney U test with Bonferroni correction between PSS (including both CMV positive and negative) and controls. The results showed that PSS aqueous had significantly higher concentrations of IL-5 (p<0.001), IL-6 (p = 0.002), CXCL8 (p<0.001), CCL2 (p = 0.002), CCL4 (p = 0.035), G-CSF (p = 0.012) and TGF-β2 (p<0.001) compared to cataract controls (Table 2).

When the PSS samples were separated into CMV+PSS and CMV-PSS groups, the increase of the above cytokines was still statistically significant in both groups compared to controls except for CCL4 (p = 0.071 CMV-PSS compared to controls, p = 0.332 CMV+PSS compared to controls).

On the other hand, the PSS group showed significantly lower median concentrations of IL-2 (p<0.001), IL-12 (p<0.001), TNF-α (p<0.001), IFN-α (p = 0.017) and GM-CSF (p = 0.005) than controls. When the PSS group was separated into CMV+ and CMV- groups, the differences on IL-2 (p<0.001), IL-12 (p<0.001), TNF-α (p<0.001) and GM-CSF (p = 0.008) remained significant between CMV-PSS and controls. Between CMV+PSS and controls, the difference on IL-2 (p = 0.026), IL-12 (p = 0.014) and TNF-α (p = 0.037) were also significant.

No significant changes on IL-4, IL-13, IL-17, IL-10, CXCL9, CXCL10 and IFN-γ levels were observed between PSS group and controls.

Between CMV+PSS and CMV-PSS groups, no significant difference on individual cytokines was observed.

**Figure 1:**
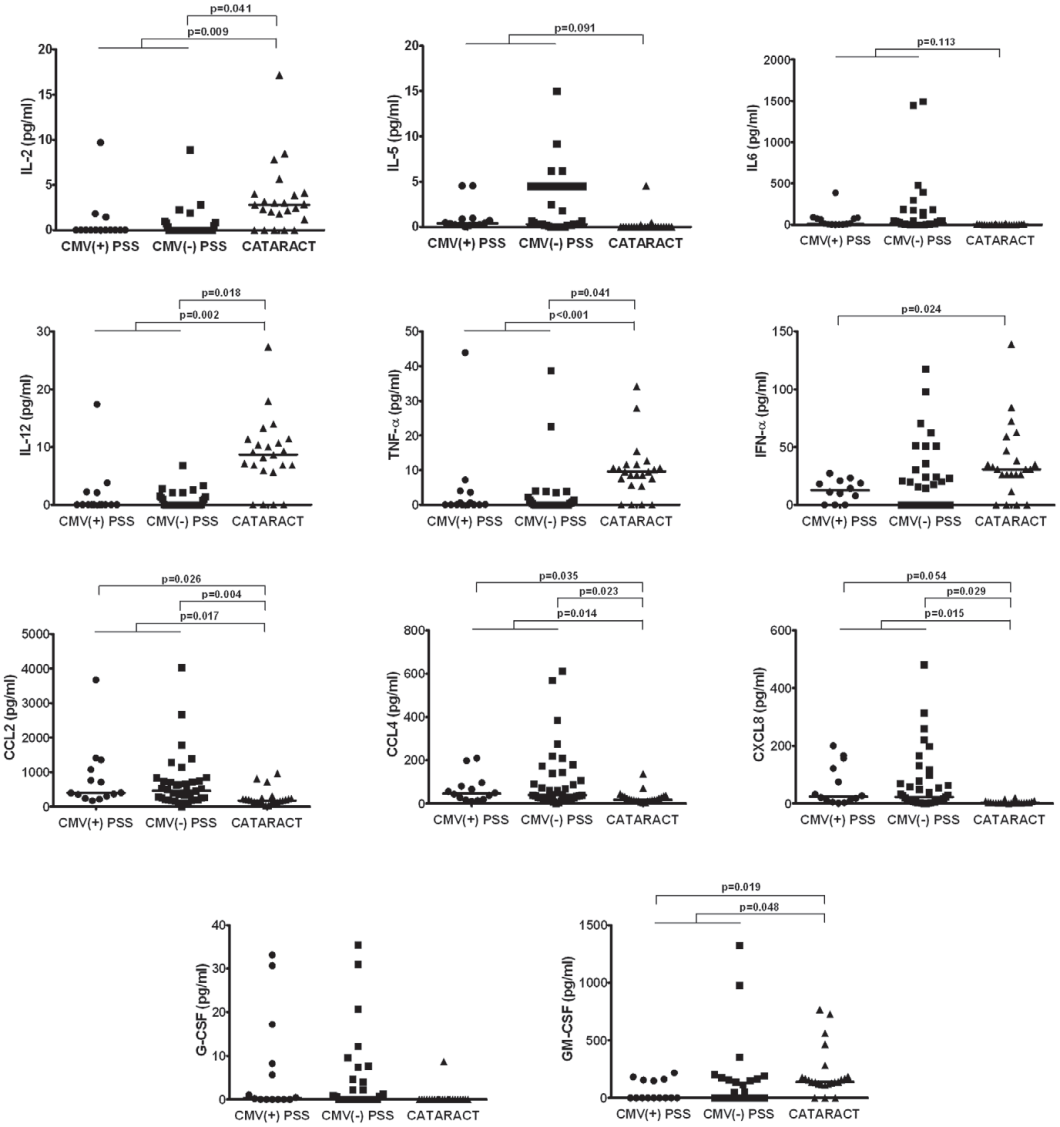
Distribution of cytokine concentrations for individual samples of CMV+PSS, CMV-PSS and control groups. The concentrations were compared between PSS (both CMV+ and CMV-) vs. controls, CMV+PSS vs. controls, and CMV-PSS vs. controls by binary logistic analysis using age and sex as variables.

Since there were significant differences in the age and gender distribution between PSS and controls groups, we further performed the binary logistic regression analysis adjusted for age and gender for each cytokine. The results were largely consistent with the Mann-Whitney U test. The differences in IL-2 (p = 0.009), IL-12 (p = 0.002), TNF-α (p<0.001), CCL2 (p = 0.017), CCL4 (p = 0.014), CXCL8 (p = 0.015), GM-CSF (p = 0.048) between PSS and controls remained significant. However, the differences in IL-5 (p = 0.091) and IL-6 (p = 0.113) was not significant anymore between the two groups. Between CMV-PSS and controls, significant difference was found in IL-2 (p = 0.041), IL-12 (p = 0.018), TNF-α (p = 0.041), CCL2 (p = 0.004), CCL4 (p = 0.023), CXCL8 (p = 0.029). Between CMV+PSS and controls, significant difference was found in INF-α (p = 0.024), CCL2 (p = 0.026), CCL4 (p = 0.035), CXCL8 (p = 0.054) and GM-CSF (p = 0.019). Figure 1 shows the concentrations of the above cytokines for each sample in the three groups.

### Classification Analysis of Cytokines between PSS and Controls

**Figure 2:**
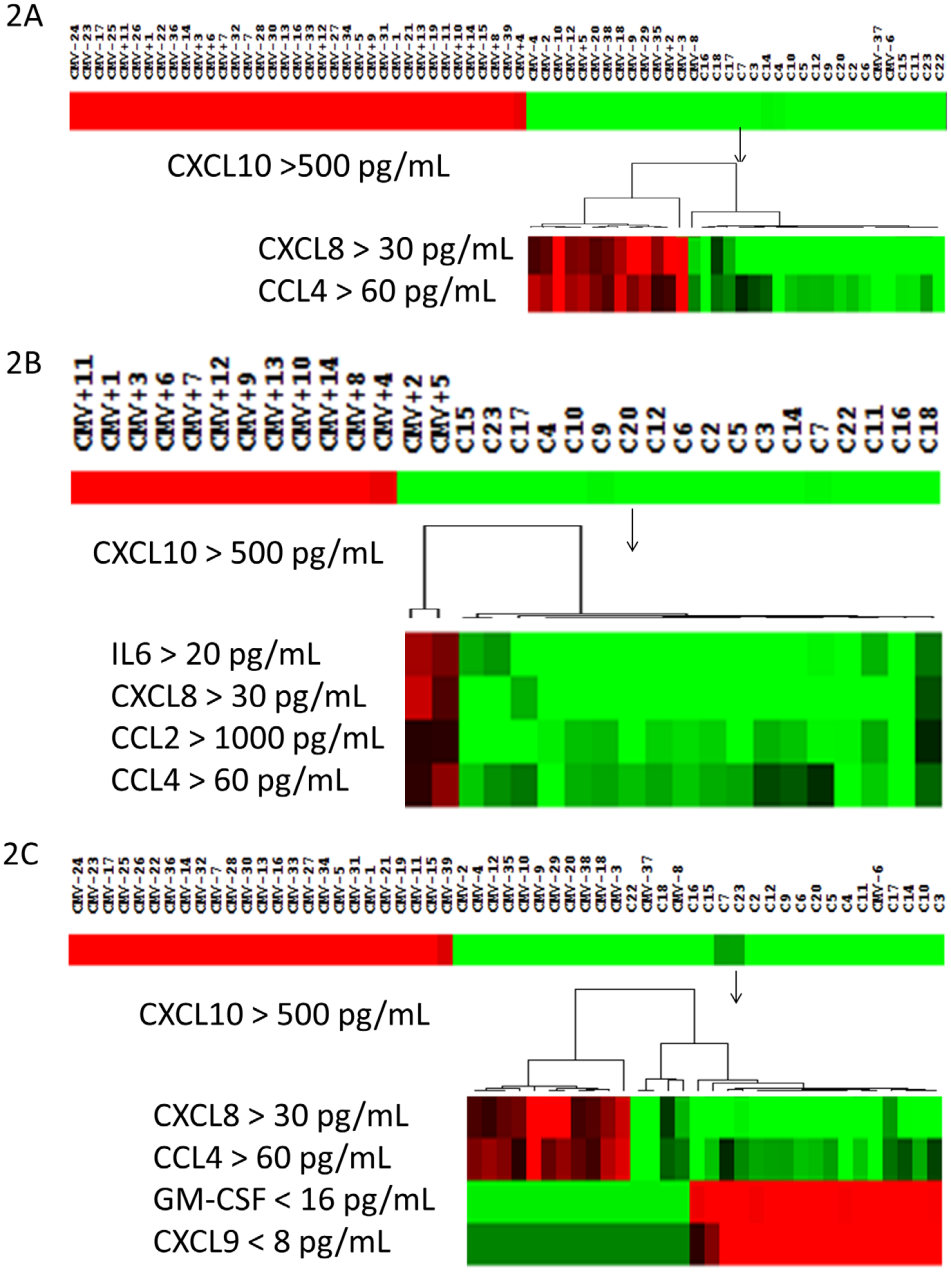
Classification of PSS, CMV+PSS and controls by stepwise heatmap analysis. Red colour indicates ‘high concentration’ and green indicates ‘low concentration’. Most of the PSS samples were separated from controls by high CXCL10 level (Figures 2A, 2B and 2C). Sixteen PSS samples (CMV- and CMV+ combined) had CXCL10 levels lower than 500 pg/mL (Figure 2A). Fourteen of them were further segregated from controls by their higher CXCL8 (>30 pg/mL) and CCL4 (>60 pg/mL) levels. Two remained in the control group. The overall accuracy was 96.3%. Twelve out of 14 CMV+PSS samples had CXCL8 exceeding 500 pg/mL (Figure 2B). The remaining 2 samples were separated from controls by their elevated IL6, CXCL8, CCL2 and CCL4 levels. The overall accuracy was 100%. Twenty-five out of 39 CMV-PSS samples had CXCL8 greater than 500 pg/mL (Figure 2C). Thirteen out of 14 remaining PSS samples were differentiated from controls by their combined elevated CXCL8, CCL4, GM-CSF and CXCL9 levels. One remained in the control group. The overall accuracy was 64% for CMV-PSS samples.

The results of classification analysis are shown as heat maps in Figure 2. In order to minimize the potential effect of age on the analysis, we excluded controls who were aged 75 and above, therefore left with 18 controls. High CXCL10 (>500 pg/mL) level separated 37 PSS samples from a total of 53 PSS (69.81%). For the rest of the PSS samples, elevated concentration of CXCL8 (>30 pg/mL) or CCL4 (>60 pg/mL) separated 14 of them from the controls (Figure 2A). However, two CMV-PSS samples remained in the control group, translating to an error rate of 3.77%.

The classification profiles were largely similar when the CMV+PSS and CMV-PSS groups were analyzed against controls separately. Twelve out of 14 CMV+PSS samples (85.71%) were separated from controls by CXCL10>500 pg/mL. The remaining 2 samples were separated from the controls by combined elevated levels of IL-6 (>20 pg/mL), CXCL8 (>30 pg/mL), CCL2 (>1000 pg/mL) and CCL4 (>60 pg/mL) (Figure 2B).

Between CMV-PSS and controls, 25 CMV-PSS were separated from controls by high CXCL10 levels (64.10%). The rest of the CMV-PSS samples were separated from controls by elevated levels of CXCL8 (>30 pg/mL), CCL4 (>60 pg/mL) with concurrent reduced concentrations of GM-CSF (<16 pg/mL) and CXCL9 (<8 pg/mL). The overall accuracy of such classification scheme for CMV-PSS patients was 92.30%, leaving 3 CMV-PSS samples in the cataract group (Figure 2C).

## Discussion

In this study, we measured the concentration of 20 cytokines in aqueous samples of 53 PSS patients, which included 14 CMV DNA positive samples and 39 CMV viral negative samples and 23 otherwise healthy cataract patients without ocular inflammatory conditions. The PSS aqueous showed significantly increased levels of CCL2, CCL4 and CXCL8; decreased levels of IL-2, IL-12, IFN-α,TNF-α and GM-CSF. PSS samples also showed higher levels of IL-6, IL-5 and G-CSF than controls when the age difference between the two groups was ignored. No significant changes of IL-4, IL-13, IL-17 and IL-10 were observed between PSS patients and cataract controls. No significant difference was observed between CMV+PSS and CMV-PSS in cytokines measured.

A number of studies have reported the changes of aqueous cytokines in different clinical entities of uveitis, including infectious uveitis [27], [28], Behcet’s disease, Vogt-Koyanagi-Harada (VKH) disease, HLA-B27-associated anterior uveitis [29], [30], Fuchs heterochromic cyclitis (FHC) and other clinically idiopathic uveitis [31]–[35]. Commonly found changes associated with intraocular inflammation regardless of disease etiology are increased IL-6, CCL2, CXCL8 levels and the switch of granulocyte stimulating factor from primary GM-CSF to G-CSF. We found similar changes of these cytokines in PSS patient samples in the present study. In addition, elevated IL-6 and CXCL8 levels were also reported in glaucomatous aqueous with elevated IOP [36], [37], suggesting that part of the inflammatory changes observed in PSS aqueous may be related to high IOP associated trabecular meshwork response.

A distinct feature of PSS aqueous is the increased CXCL10 (>500 pg/mL), CCL4 (>60 pg/mL) and CXCL8 (>30 pg/mL) levels as revealed by the classification analysis. Additional factors, such as CCL2, IL-6, GM-CSF and CXCL9 were included in the cluster to better separate CMV-PSS and CMV+PSS samples from controls. Among these cytokines, CXCL10 is the most powerful factor which separated 70% of all PSS samples, even though statistical analysis showed no significant difference between the groups largely due to big variations. CCL2, CCL4 and CXCL8 are chemokines involved in neutrophil and monocyte recruitment. CCL2, CCL4 and CXCL10 also regulate the polarization of T helper cells into Th1 *versus* Th2 cells. However, other than IL-6 and IL-5, no significant increase of other proinflammatory cytokines were observed in PSS samples compared to controls. When compared to infectious uveitis aqueous samples, the increase of the above chemokines in PSS samples were small (Li et al, unpublished data). Consistently, few infiltrated leukocytes were observed in the anterior chamber of PSS patients. Collectively, these changes depicted a mild inflammatory profile associated with PSS, rather than infective even in CMV+PSS eyes. It suggests that even though PSS may be triggered by CMV in the majority of eyes [15], the clinical features are a manifestation of predominantly immune mediated inflammatory changes. This may also account for the recurrent nature of the disease and the response to anti-inflammatory therapy such as topical nonsteroidal antiinflammatory drugs (NSAIDs).

While the existence of CMV DNA in the aqueous of immunocompetent uveitis patients have been reported by several groups, the source and the effect of aqueous CMV on intraocular inflammation remain unknown. As far as we are aware, this is the first study which measured a comprehensive range of cytokines in the aqueous of immunocompetent uveitis patients who are positive of CMV DNA and our results showed no significant difference in aqueous cytokine concentrations between CMV+ and CMV-PSS patients. This supports the theory that even if CMV is not detected in the aqueous of all PSS patients, the cytokines expressed suggest a common final pathway of immune-mediated inflammation.

There are a few possible explanations for the lack of differences observed: The number of CMV in the aqueous may not be enough to cause significant changes in aqueous cytokines during the episode. CMV is also known to evade both innate and adaptive immune systems by multiple mechanisms involving the down regulation of MHC expression, inhibition of dendritic cell and natural killer cell activation and chemokine sequestration. [2] Furthermore, the lack of difference may indicate that a transient intraocular CMV infection/reactivation occurred in all PSS patients. However, in CMV-PSS patients the infection was controlled therefore the aqueous CMV DNA at the time of sampling was negative, while in CMV+PSS patients the infection persisted. Ulcerative colitis is another disease which is often associated with CMV reactivation in immunocompetent patients. [4] CMV reactivation was more extensively studied in colitis and a 20–80% CMV DNA positive rate was reported in these patients. However, the effect of human CMV reactivation on colitis remains contradictory. [38] Clearly further studies are needed to determine the effect of intraocular CMV infection/reactivation on the precipitation and aggravation of intraocular inflammation.

The study has limitations. The control patients were significantly older than the PSS patients and the sex distribution in the PSS group was not even. The effect of age and gender on individual cytokine is unknown. Our analysis indicated that IL-6 and IL-5 levels may be related to the age of donors. Another limit of the study is the sample size, especially in the CMV+PSS group. This is largely due to the lack of aqueous samples. In fact more CMV+PSS patients were identified during the study period, however only a small percentage of these patients had enough aqueous samples available for the cytokine measurement. This is because extra aqueous samples were needed for quantitative PCR analysis on CMV+ samples. For future studies, we suggest combining aqueous samples taken from patients of the same disease group for similar analysis.

In conclusion, our study showed that the inflammatory changes in PSS aqueous are largely featured by increased chemokines such as CXCL8, CXCL10, CCL4 and CCL4. Our results also showed that the presence of CMV viral DNA in the aqueous did not cause significant changes in aqueous cytokine concentrations in immunocompetent PSS patients. However, further studies are required to discern whether CMV is an innocent “bystander” or the culprit of the inflammation observed in these patients.
